# The Treatment of Facial Neuralgia by Antipyrine

**Published:** 1888-02

**Authors:** 


### The Treatment of Facial Neuralgia by Antipyrine.

One by one the non-inflammatory painful affections are wheeling into line as amenable to treatment by antipyrine. Germain See, in speaking of this subject, says: "To complete the series of painful affections of the head which have yielded to antipyrine, I must mention facial neuralgia. I have notes of seven cases of tic doulоureux, all of a very grave kind, two of which were completely cured. One resisted antipyrine absolutely, while four have experienced marked amelioration and appear to be on the way to recovery. These patients had been suffering from tic douloureux from twelve to eighteen years. During this long and frightful period of suffering, these patients had never been able, without pain, to open their mouths, to speak, to chew their food, to swallow hot or cold liquids, to expose themselves to a current of air, or to enjoy the least respite, even under the influence of morphia or salycilate of soda. These four patients are enabled now, after two months of treatment, to enjoy that freedom from pain which they had not before known for years, and to live like the other members of the family. The treatment has consisted in the daily use of 75 grains of antipyrine (15 grains every four hours, til the entire quantity was taken). I have also relied very much on the subcutaneous injections of antipyrine--8 grains dissolved in the same weight of water, and the whole injected for one dose--but as these injections have sometimes been painful, I have lately modified my formula, as follows: I now dissolve 8 grains of antipyrine in 22 grains of water, to which, in order to enhance the effect, I sometimes add $\frac{1}{6}$ grain of cocaine. These injections act with surprising rapidity and energy. I now rely on these hypodermic injections in all inveterate cases, and during the painful paroxysms combine the hypodermic treatment with the internal administration of 75 grains a day. The results in this most grave and most intractable of painful disorders have been unprecedentedly gratifying and surprising."

---

Cracks in Floors.—A very complete filling for open cracks in floors may be made by thoroughly soaking newspapers in paste made of one pound of flour, three quarts of water, and a teaspoonful of alum, thoroughly boiled and mixed. Make the final mixture about as thick as putty, and it will harden like papier mache. This paper may be used for molds for various purposes. —*Cal. Architect.*

